# Transcranial magnetic stimulation in the treatment of phantom limb pain: a systematic review

**DOI:** 10.1055/s-0044-1779051

**Published:** 2024-01-29

**Authors:** Gabriel Rocha Santos Knorst, Phamella Rocha de Souza, Armani Gontijo Plácido Di Araújo, Samantha Avanço Ferraz Knorst, Denise Sisterolli Diniz, Hélio Fernandes da Silva Filho

**Affiliations:** 1 Universidade Federal de Goiás, Hospital das Clínicas, Departamento de Neurologia, Goiânia GO, Brazil.; 2 Pontifícia Universidade Católica de Goiás, Goiânia GO, Brazil.

**Keywords:** Phantom Limb, Transcranial Magnetic Stimulation, Pain, Systematic Review, Membro Fantasma, Estimulação Magnética Transcraniana, Dor, Revisão Sistemática

## Abstract

**Background** Phantom limb pain (PLP) occurs after amputations and can persist in a chronic and debilitating way. Repetitive transcranial magnetic stimulation (rTMS) is a non-invasive neuromodulation method capable of influencing brain function and modulating cortical excitability. Its effectiveness in treating chronic pain is promising.

**Objective** To evaluate the evidence on the efficacy and safety of using rTMS in the treatment of PLP, observing the stimulation parameters used, side effects, and benefits of the therapy.

**Methods** This is a systematic review of scientific articles published in national and international literature using electronic platforms.

**Results** Two hundred and fifty two articles were identified. Two hundred and forty six publications were removed because they were duplicated or met the exclusion criteria. After selection, six studies were reviewed, those being two randomized clinical trials and four case reports. All evaluated studies indicated some degree of benefit of rTMS to relieve painful symptoms, even temporarily. Pain perception was lower at the end of treatment when compared to the period prior to the sessions and remained during patient follow-up. There was no standardization of the stimulation parameters used. There were no reports of serious adverse events. The effects of long-term therapy have not been evaluated.

**Conclusion** There are some benefits, even if temporary, in the use of rTMS to relieve painful symptoms in PLP. High-frequency stimulation at M1 demonstrated a significant analgesic effect. Given the potential that has been demonstrated, but limited by the paucity of high-quality studies, further controlled studies are needed to establish and standardize the clinical use of the method.

## Resumo

**Antecedentes** A dor do membro fantasma (DMF) ocorre após amputações e pode persistir de forma crônica e debilitante. A estimulação magnética transcraniana repetitiva (EMTr) é um método de neuromodulação não invasivo capaz de influenciar a função cerebral e modular a excitabilidade cortical. Sua eficácia no tratamento da dor crônica é promissora.

**Objetivo** Avaliar as evidências sobre a eficácia e segurança do uso da EMTr no tratamento da DMF, observando os parâmetros de estimulação utilizados, efeitos colaterais e benefícios da terapia.

**Métodos** Trata-se de uma revisão sistemática de artigos científicos publicados na literatura nacional e internacional utilizando plataformas eletrônicas.

**Resultados** Foram identificados 252 artigos. Duzentas e quarenta e seis publicações foram removidas por estarem duplicadas ou atenderem aos critérios de exclusão. Após a seleção, foram revisados seis estudos, sendo dois ensaios clínicos randomizados e quatro relatos de caso. Todos os estudos avaliados indicaram algum grau de benefício da EMTr no alívio dos sintomas dolorosos, mesmo que temporariamente. A percepção da dor foi menor ao final do tratamento quando comparada ao período anterior às sessões e permaneceu durante o acompanhamento do paciente. Não houve padronização dos parâmetros de estimulação utilizados. Não houve relatos de eventos adversos graves. Os efeitos da terapia a longo prazo não foram avaliados.

**Conclusão** Existem alguns benefícios, mesmo que temporários, no uso da EMTr para alívio dos sintomas dolorosos na DMF. A estimulação de alta frequência em M1 demonstrou um efeito analgésico significativo. Dado o potencial demonstrado, mas limitado pela escassez de estudos de alta qualidade, são necessários mais estudos controlados para estabelecer e padronizar o uso clínico do método.

## INTRODUCTION

Pain is currently defined by the International Association for the Study of Pain (IASP) as “an unpleasant sensory and emotional experience associated with tissue damage real or potential” 1 . According to Melzack and Katz, pain is “a personal, subjective experience influenced by cultural learning, the meaning of the situation, attention, and other psychological variables” 2 . Pain cannot be considered a simple product of a linear transmission of nerve impulses. Rather, it is a dynamic process involving continuous interactions among complex ascending and descending systems. 2

Chronic pain is highly prevalent worldwide and has been acknowledged as a major public health problem in many countries. Pain affects 20% to 40% of the general population in Latin America and has known associations with depressed mood, fatigue, and catastrophizing thoughts. It is not a unique disease, but there are different pain syndromes with different treatments and response rates. 3

Over the years, several theories have tried to explain the origin, mechanisms, and consequences of pain. First, was published the direct-line sensory projection system called Descartes' specificity theory, in which large and small fibers were assumed to transmit touch and pain impulses, in separate and specific pathways to touch and pain centers in the brain. Years later, the gate control theory of pain was created, the first to incorporate the central control processes of the brain. According to this theory, the transmission of nerve impulses from afferent fibers to spinal cord transmission cells is modulated by a gating mechanism in the spinal dorsal horn and influenced by nerve impulses that descend from the brain. 2

There are three phenomena that often coexist in the same individual postamputation. They are phantom sensations, stump pain, and phantom limb pain. Phantom sensations are nonpainful perceptions arising from the lost body part after deafferentation or amputation. They are experienced very frequently immediately after amputation but decrease with time and can be a kind of kinesthetic feelings of length, volume, or other spatial sensations of the amputated limb. On the other hand, stump pain, also known as residual limb pain, is perceived in the amputation stump or residual limb. It is frequent immediately after amputation, but chronic stump pain can occur in 5-10% of all amputees. 4 5 Lastly, phantom limb pain (PLP) is a type of pain that occurs in a limb that no longer exists. 6

PLP is very common in the early stages after a limb amputation, but it can persist chronically for many years. It is estimated that it occurs in about 50 to 80% of cases, and 5 to 10% of these experience extreme pain. 7 8 It is usually characterized in different ways, such as sharp pain, shooting, electric, dullness, tightness, and colic. 6 It represents an example of deafferentation pain and is considered a public health problem because it affects not only the physical health of amputees but also compromises their psychological and functional health. 6 7 In the long term, PLP can directly interfere with quality of life and lead to other comorbidities such as depression, sleep disorders, and substance abuse. 6

The pathophysiology of PLP is not yet fully established. It is believed to be related to alterations in the reorganization of the somatosensory and motor cortices resulting from the amputation. 6 7 9 But, as we could see, pain is also felt in the absence of inputs from the body and probably the origin of the patterns of this experience lies in neural networks in the brain. In amputated patients, sensory inputs merely modulate the phantom experience, however, they do not directly cause it. 2

The vision of the body as a unity, with different qualities at different times, is part of a new theory called Neuromatrix. According to this theory, the neuromatrix comprises a widespread network of neurons that generate patterns, process information and produce the multiple dimensions of pain experience, as well as concurrent homeostatic and behavioral responses. The neuromatrix theory of brain function, can be the basis of phantom limb phenomena, providing an explanation for phantom limb pain. 2

Traditionally, PLP is being considered a complex chronic pain syndrome that is difficult to treat, responding poorly to conventional therapies. 6 7 8 The treatment of PLP includes pharmacological control, non-pharmacological invasive and non-invasive strategies. 8 However, there is inconclusive evidence in the literature for any single therapy and none has proved to be particularly efficacious. 2 10

Neuromodulation involves techniques for assessing and treating of the neurological tissue, both centrally and peripherally. It can be invasive and non-invasive. Targeting phantom limb pain, transcranial magnetic stimulation (TMS) is one of these non-invasive neuromodulation methods capable of studying plasticity patterns and cortical reorganization, dictating the parameters to be used to alter the maladaptive neuroplasticity and increase the descending inhibitory pathways. 6 When stimulation is performed repeatedly (rTMS) it is able to influence brain function, modulating cortical excitability, being a potential tool for the treatment of this chronic pain. 7

Studies, however, that evaluated the therapeutic utility of rTMS in the management of PLP are limited and have divergent evidence. Some have noted benefits in reducing phantom pain, increasing serum beta-endorphin production, and reducing anxiety and depressive symptoms after treatment. 6 7 8 10 Others showed no significant difference when compared with the placebo group or the benefit ceased to be significant after 30 days of stimulation. 6 10

The aim of this study is to systematically review the evidence on the efficacy and safety of rTMS in the treatment of phantom limb pain, observing the stimulation parameters used, side effects, and therapy benefits.

## METHODS

### Type of study

This is a systematic review of scientific articles published in national and international literature. The guidelines contained in the Cochrane manual for systematic reviews of interventions 11 and in the PRISMA statement 12 were used as references for the development and reporting of the study **.**

### Eligibility criteria

The criteria adopted for the selection of studies in this review were the following: Studies that used TMS in the treatment of adults over 18 years of age diagnosed with PLP after amputation for any reason; Randomized and non-randomized clinical trials, case reports; Publications with full text in Portuguese or English.

Exclusion criteria were: studies that used invasive methods of central nervous system stimulation, peripheral nervous system stimulation, or other non-invasive neuromodulation methods other than TMS; articles that addressed a topic unrelated to the purpose of the study; articles with incomplete data that made evaluation and comparison with other studies impossible; chronic pain unrelated to amputation; literature reviews; articles that did not address treatment with TMS; incomplete publications or articles published in languages other than Portuguese or English.

The assessment of pain severity was chosen as the primary outcome. It is usually considered significant pain relief when there is an improvement of at least 30% or 2/10 on the Visual Analog Pain Scale. 9 13 There were no restrictions for secondary outcomes, which may be related to adverse effects and stimulation safety as well as the impact on the biopsychosocial context of patients such as quality of life, mood, depression, and anxiety.

### Information sources

The literature search was carried out using the electronic platforms: PubMed, Embase, Cochrane Library, Scientific Electronic Library Online (SciELO), and Latin American and Caribbean Literature in Health Sciences (LILACS) to search for scientific articles published until October 2022.

### Search strategy

After consulting the DeCS and MESH platforms, the following descriptors were chosen: "phantom limb" and "transcranial magnetic stimulation". The Boolean operator "AND" was used in the search system to link the terms.

### Selection of studies

Based on the results obtained, the title and abstract of the studies found were analyzed by two independent reviewers to assess whether or not they were appropriate for the research object.

**Figure 1:**
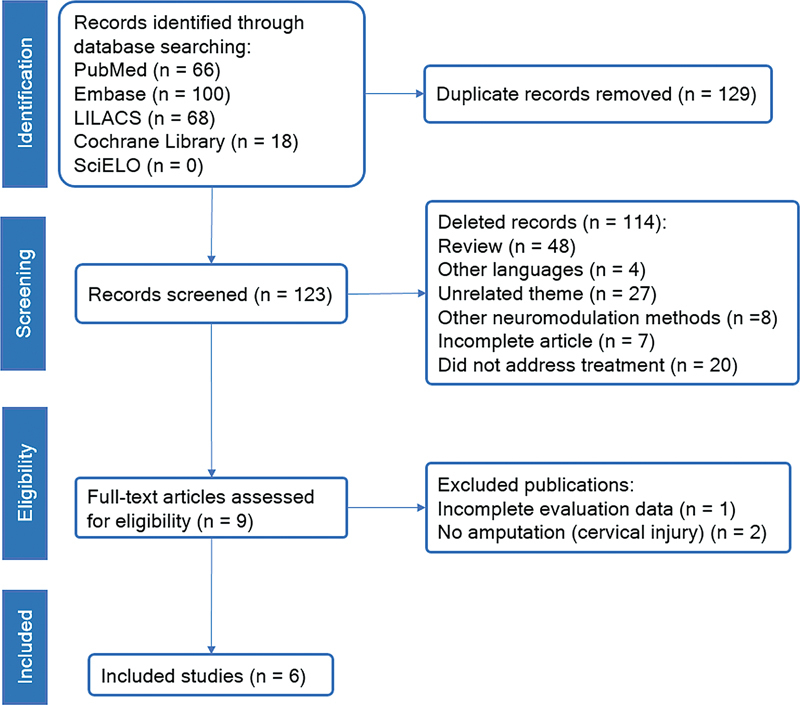
PRISMA flow diagram of the study selection process.

Articles that focused on the treatment of phantom limb pain after amputation using TMS were fully evaluated and their data were extracted using a standardized form among researchers in order to document the following information: sample characteristics, study design, intensity of PLP, quality of life, efficacy, safety and adverse events of TMS, as well as frequency of stimulation, cortical region stimulated, duration of sessions and duration of effects of stimulation after treatment in these patients. Review articles, duplicate articles, and unrelated articles were excluded ( Figure 1 ).

## RESULTS

Two hundred fifty-two articles were identified in the databases. These articles underwent to manual review process by the researchers and 129 were removed from the screening because they were duplicated, remaining 123 articles. These articles had their titles and abstracts evaluated by three independent researchers and 114 publications were removed from the study because they met the exclusion criteria.

The remaining 9 articles had their full texts evaluated individually by the researchers regarding the eligibility criteria. 3 of these articles were excluded at this stage, 2 of them due to the use of the term phantom pain in contexts in which there was no limb amputation, and 1 due to the lack of sufficient data necessary for the evaluation and comparison between the publications of this study.

After selection, 6 articles were included for final analysis, namely: Ahmed, 14 Malavera, 15 Scibilia, 16 Grammer, 17 Lee, 18 and Di Rollo. 19 The entire study selection process is described in Figure 1 in the flow diagram suggested by the PRISMA statement.

### Study characteristics

The main characteristics of the studies in this review are summarized in Table 1 . Of the 6 selected articles, 2 were randomized clinical trials and 4 were case reports. A total of 85 adults with clinical symptoms of phantom limb pain after amputation were included in this review, 73 men and 12 women. The mean age was 56.3 years.

**Table 1:** Characteristics of rTMS studies in patients with PLP

References	Type of study	Participants (n, age, sex)	Side, level, and reason of amputation	Duration (months since amputation)	Outcome measurements	Follow-up	Conclusion
Ahmed et al. (2011)	RCT	EG: n = 17 / Age: 51.01 ± 12.7 / Sex: 13 male / 4 female	Side: not mentioned // Level: UL = 7 (EG) e 4 (CG) / LL = 10 (EG) e 6 (CG) // Reason of amputation: traumatic = 13 / Ischemic = 6 / Diabetic = 8	EG: 33.4 ± 39.3	VAS, LANSS, HAM-D, HAM-A	Baseline, after the 1st session, after the 5th session, 1 month and 2 months after the last session	rTMS provided lasting relief from PLP for at least 2 months. The rTMS increased serum Betaendorphin levels in the CNS
		CG: n = 10 / Age: 53.03 ± 13.3 / Sex: 6 male / 4 female		CG: 31.9 ± 21.9			
Di Rollo et al. (2011)	Case report	n = 1 / Age: 36 / Sex: male	Side: left / Level: UL / Reason of amputation: traumatic	120	VAS, HAM-D, HAM-A, MRS, CORSI TEST, PVF	Weekly for 6 weeks	rTMS improved PLP with lasting analgesic effects after 3 weeks
Malavera et al. (2016)	RCT	EG: n = 27 / Age: 33.1 ± 6.6 / Sex: 25 male / 2 female	Side: not mentioned/ Level: LL = 54 / Reason of amputation: traumatic	EG: 683.76 ± 67.2	VAS, SDS, SAS	1 week before treatment, 15 days and 30 days after treatment	rTMS induced significant clinical improvement in PLP up to 15 days after treatment
		CG: n = 27 / Age: 34.7 ± 9.9 / Sex: 25 male / 2 female		CG: 98.4 ± 75.6			
Scibilia et al. (2018)	Case report	n = 1 / Age: 69 / Sex: male	Side: right / Level: LL / Reason of amputation: traumatic	480	VAS	Baseline, after each session, 1 month and 6 months after treatment	rTMS induced marked and lasting cortical and subcortical plasticity associated with pain reduction
Grammer et al. (2015)	Case report	n = 1 / Age: 24 / Sex: male	Side: right / Level: UL / Reason of amputation: traumatic	5	VAS	Baseline, after the 4th session and after the 28th session (6 weeks)	rEMTR may be effective for upper limb PLP
Lee et al. (2015)	Case report	n = 1 / Age: 37 / Sex: male	Side: right / Level: LL / Reason of amputation: neoplasm	96	VAS, BDI, BFI	Baseline, after each session and 3 months after treatment	rTMS resulted in a dramatic decrease in PLP, with an analgesic effect that lasted for about 3 months.

Abbreviations: BDI, Beck Depression Inventory; BFI, Brief Fatigue Inventory; CG, control group; EG, experimental group; HAM-A, Hamilton Rating scale for Anxiety; HAM-D, Hamilton Rating Scale for Depression; LANSS, Leeds Assessment of Neuropathic Symptoms and Signs Pain Scale; LL, lower limb; MRS, Mania Rating Scale; PLP, phantom limb pain; PVF, Phonemic Verbal Fluency; RCT, randomized controlled trial; rTMS, repetitive transcranial magnetic stimulation; SAS, Zung Self-Rating Anxiety Scale; SDS, Zung Self-Rating Depression Scale; UL, upper limb; VAS, Visual Analog Scale.

With regard to data related to amputation, all articles distinguished the affected limb between upper and lower limbs, making a total of 13 participants with upper limb amputation and 72 participants with lower limb amputation. The 2 clinical trials Ahmed 14 and Malavera 15 did not mention the amputated side. Participants in the studies of Scibilia, 16 Grammer 17 and Lee 18 underwent amputation on the right side, while in Di Rollo 17 the amputation occurred on the left side. There were several causes of amputation reported in the studies, with traumatic etiology being the most common (n = 70), followed by diabetes (n = 8), ischemia (n = 6), and neoplasia (n = 1).

The duration between the amputation date and the start of stimulation sessions was extremely variable between studies. The minimum duration was 5 months in Grammer, 17 while the maximum duration was 57 years in Malavera, 15 with an average time of 29.54 months.

The patients were evaluated before starting the treatment (baseline assessment), after the sessions, and in some cases, such as Ahmed, 14 Di Rollo, 19 Malavera 15 and Lee, 18 the follow-up of the patients was maintained for a period after the end of the treatment to assess the lasting effects. of therapy. The minimum follow-up time was 30 days and the maximum was 6 months, with an average of approximately 10 weeks.

All 6 studies assessed pain intensity before and after treatment using the Visual Analog Scale (VAS). Ahmed 14 also applied the Leeds Assessment of Neuropathic Symptoms and Signs Pain Scale (LANSS) used to assess general neuropathic pain.

Other studies intended to assess psychological symptoms such as anxiety, depression, and cognitive changes. For that, they used the following instruments: Brief Fatigue Inventory (BFI) and Beck Depression Inventory (BDI) in Lee 18 ; Zung Self-Rating Depression Scale (SDS) and Zung Self-Rating Anxiety Scale (SAS) in Malavera 15 ; Hamilton Rating Scale for Depression (HAM-D), Hamilton Rating scale for Anxiety (HAM-A), Mania Rating Scale (MRS), CORSI TEST and Phonemic Verbal Fluency (PVF) in Di Rollo. 19

### Stimulation parameters

The main TMS parameters used to treat phantom limb pain in the evaluated studies are summarized in Table 2 . They were target, frequency, intensity, number of pulses, and number of sessions.

**Table 2:** rTMS parameters in patients with PLP

References	Stimulation target	Stimulation frequence (Hz)	Stimulation intensity (% LMR)	Number of pulses (n)	Number of sessions (n)
Ahmed et al. (2011)	M1 contralateral	20	80	200	5
Di Rollo et al. (2011)	M1 ipsilateral	1	80	600	15
Malavera et al. (2016)	M1 contralateral	10	90	1200	10
Scibilia et al. (2018)	M1 e DLPFC contralateral	10	120	3000	30
	PSA contralateral	1	100	2002	30
Grammer et al. (2015)	PSA contralateral	1	100	2000	17
	DLPFC contralateral	10	120	3000	11
Lee et al. (2015)	M1 contralateral	1	85	800	10
	SMC contralateral	1	85	800	50

Abbreviations: DLPFC, dorsolateral prefrontal cortex; M1, motor cortex; PLP, phantom limb pain; PSA, primary sensory area; RMT, resting motor threshold; rTMS, repetitive transcranial magnetic stimulation; SMC, supplementary motor complex.

### Stimulation target

The majority of studies used the contralateral motor cortex (M1) to the amputated side as a target. In three studies, only a single target (M1) was used ( 14 15 19 ), while in the rest of the evaluated studies, at least 2 different targets were used, among them the dorsolateral prefrontal cortex (DLPFC) ( 16 17 ), the primary sensory area (PSA) ( 16 17 ) and the supplementary motor complex (SMC) ( 18 ).

### Stimulation frequency

Stimulation frequency is an important parameter used in TMS. It is usually divided into two groups: high-frequency, considered excitatory and comprised in the range of 5 to 20Hz, and low frequency, considered inhibitory and comprised in the range less than or equal to 1Hz. Among the evaluated studies, high-frequency stimulation was used exclusively in two, one with 20 Hz and the other with 10 Hz. Low-frequency stimulation was used exclusively in one study. Four other studies used combinations of high and low frequency in different periods and cortical targets, as described in Table 2 .

### Stimulation intensity

Stimulation intensity is individually calculated from the first session after establishing the Resting Motor Threshold (RMT), which is defined as the minimum stimulator intensity necessary to evoke at least one visible muscle contraction in the extensor hallucis brevis muscle, while maintaining a relaxed position. 20 After establishing the patient's threshold, the intensity is defined from a percentage on top of the MRL value, depending on the patient's tolerance. Among the 6 studies, 2 chose intensity 80% of the RMT, 1 chose 85% of the RMT, 1 chose 90% of the RMT and 2 studies chose from 100% to 120% of the RMT.

### Number of pulses

Pulses in TMS can be divided into single pulse, paired pulse, and repetitive pulse (rTMS). Repetitive pulse stimulation is the most used for analgesia purposes. The reviewed studies used an extremely variable number of pulses, as follows: 2 studies between 2000-3000 pulses, 1 study 1200 pulses, 1 study 800 pulses, 1 study 600 pulses, and another study 200 pulses.

### Analgesic effect

**Figure 2:**
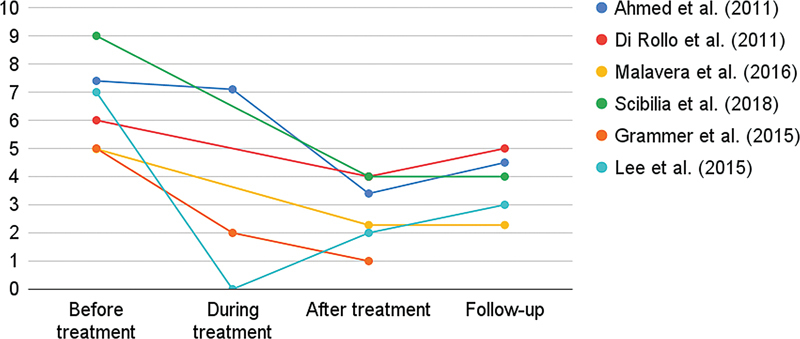
Analgesic effect of rTMS on phantom limb pain. Based on patient-reported visual analogue scale (VAS) score. The scale ranges from 0 to 10, with higher scores indicating greater severity of symptoms.

All studies reviewed indicated some benefit in the use of rTMS to relieve painful symptoms in patients with PLP. Response to treatment was measured through successive assessments using the VAS in the pre-treatment, during-treatment, and post-treatment periods. There was great variation in the follow-up of the patients. Most studies only evaluated the short-term analgesic response, which demonstrated a significant benefit in the maintenance of the reduction of pain after the end of the sessions. Only one study 16 evaluated the effect after 6 months of the sessions, and it was not possible to estimate the real effect of the therapy over time. This information is summarized in Figure 2 and Table 3 .

**Table 3:** Analgesic effect of rTMS along the treatment.

References	Analgesic effect			
	Before treatment	During treatment	After treatment	Follow-up
Ahmed et al. (2011)	7.4	1st session: 7,1	5th Session: 3.4	2 months: 4.5
Di Rollo et al. (2011)	6	−	15th Session: 4	3 weeks: 5
Malavera et al. (2016)	4.98	−	10th Session: 2.28	15 days: 2.28
Scibilia et al. (2018)	9	−	30th Session: 4	6 months: 4
Grammer et al. (2015)	5	4th Session: 2	28th Session: 1	−
Lee et al. (2015)	7	3rd Session: 0	60th Session: 2	3 months: 3

Abbreviation: rTMS, repetitive transcranial magnetic stimulation. Note: *Based on patient-reported visual analogue scale (VAS) score. The scale ranges from 0 to 10, with higher scores indicating greater severity of symptoms.

### Secondary outcomes

#### *Adverse effects*

Regarding stimulation-related adverse effects, Lee 18 reported blurred vision in one eye with complete recovery after treatment. In Malavera, 15 no serious adverse effects were reported. Some patients experienced mild effects such as headache (11.1%), neck pain (5.5%), and drowsiness (18.5%) with no significant difference between groups. No adverse effects were reported in studies by Ahmed, 14 Grammer, 17 Di Rollo 19 and Scibilia. 16

#### *Emotional effects*

Ahmed 14 used the Hamilton depression and anxiety scales and demonstrated a significant decrease in patients who received real stimulation when compared to sham stimulation. In the case report by Di Rollo, 19 Hamilton's depression and anxiety scales remained stable with a score ≤ 6, showing no changes in the patient's mood after treatment. The report in Lee 18 described that the treatment led to a reduction in the score of depressive symptoms in the application of the Beck Depression Inventory (score from 28 to 8). On the other hand, Malavera 15 evaluated the influence of rTMS on anxiety and depression symptoms using the respective Zung scales and found no statistically significant difference between groups when comparing the scales on the 15th or 30th day after treatment. Studies by Grammer 17 and Scibilia 16 did not assess these parameters.

## DISCUSSION

Non-invasive brain stimulation has been extensively studied over the past 30 years to control chronic pain. 3 TMS is a non-invasive neuromodulation method whose effectiveness in treating chronic pain has been investigated. Actually, rTMS has been proposed in the treatment of chronic neuropathic or non-neuropathic pain, although the underlying pathophysiological mechanisms are different. According to the evidence-based guidelines on the therapeutic use of rTMS, 13 high-frequency stimulation of the M1 region contralateral to the painful side has a well-defined analgesic effect (Level A), while low-frequency stimulation of the M1 region contralateral to the painful side is likely to be ineffective (Level B). There were no recommendations for different cortical targets and there were no specific recommendations for PNP in this guideline.

The Latin American and Caribbean consensus on noninvasive central nervous system neuromodulation for chronic pain management 3 reviewed 22 rTMS studies and provided some recommendations: level A recommendation for high-frequency rTMS over M1 for fibromyalgia and neuropathic pain, and level B for myofascial or musculoskeletal pain, complex regional pain syndrome, and migraine. Considering other targets, the consensus recommended against the use of high-frequency rTMS over the left DLPFC in the control of pain (level B). It should be noted that there were no specific recommendations for PLP in this consensus.

The efficacy of rTMS is well established (level A of evidence) for some comorbidities that can be linked to chronic pain, such as, for example, its antidepressant effect when performed at high-frequency in the left DLPFC, as well as a probable efficacy (level B), when performed at a low frequency on the right DLPFC. 13

In the twenty-two studies of the Latin American and Caribbean consensus, 3 rTMS was more frequently administered using superficial coils targeting M1, at high frequency (10–20 Hz) in sessions comprising 1500 to 3000 pulses. However, only two studies investigated PLP 14 15 , which are the same studies present in this review.

The use of high-frequency rTMS on M1 had already been evaluated by Hirayama. 21 In this study, 20 patients with intractable deafferentation pain were treated by rTMS. The only target that had a significant effect on pain was M1 (p < 0.01). A significant reduction in pain was observed for 3 h (p < 0.05). The other stimulated targets (S1, premotor area, or SMA) were ineffective in the treatment of chronic pain. 21

It should be noted that in the Hirayama study, there were no amputee patients and that the analgesic effect was only evaluated in the short term, given that the patients received a total of 500 stimulations from each of the targets, applied once in 2 days, with time intervals about 48 h, it is not possible to predict the residual analgesic effect over time. 21

It was noted that the stimulation parameters used in the studies of this review were extremely variable, with regard to the target, frequency, intensity, and number of pulses in each stimulation. Although in the 6 studies the analgesic response was positive at the end of treatment, it is not possible to establish a standardized indication.

High-frequency stimulation of the contralateral M1 motor cortex was the most used parameter in this review (referências). This application is in accordance with international recommendations, although other targets have also been used, such as the DLPFC, PSA, SMC, and the ipsilateral M1 cortex itself subjected to stimulation. inhibitory, all with favorable results. However, due to the design of these studies and the limited sample size, there is not enough strength for a formal recommendation.

PLP is complex and its pathophysiology is still not completely understood, although its existence has been described since 1551. 9 Many theories have been raised to explain PLP, the strongest of which is the “theory of maladaptive plasticity”, which consists of the cortical reorganization after amputation and that it is not limited only to the sensorimotor cortex. 22 However, recent studies suggest that there is a significant reduction in interhemispheric functional connectivity in amputee patients and that this is associated with chronic pain. 23 The rTMS can help to reorganize the cortical mapping and increase the relationship between brain areas, a fact once reported 13 and demonstrated in the study by Scibilia, 16 in which there was an increase in the relationship between the left postcentral gyrus and other distant areas of the brain associated with a reduction in pain.

There was no separation between the studies evaluated regarding the amputated side, the cause of the amputation, and the duration of the injury. Thus, it is not possible to conclude the real participation of these factors in the genesis of PLP and in the response to treatment.

It is important to report that all studies in this review indicated some benefit in the short term of rTMS to relieve painful symptoms in patients with PLP. According to the patient's perception of pain was lower at the end of the treatment when compared to the period prior to the sessions. In all of them, there was some degree of temporary pain relief during the follow-up of these patients. The studies focused on the immediate effects of rTMS on pain relief and maintenance of relief in the short and medium term (the follow-up interval between studies was from 30 days to 6 months). However, there was no follow-up of the patients for a period greater than 6 months, and it was not possible to assess the effects of long-term therapy.

As in other reviews and randomized clinical trials, the VAS was used as a baseline pain assessment instrument and in the follow-up of patients after stimulation in all studies in this review. Because it is easy to apply and measure, the VAS proved to be a good parameter to be used and its use should be encouraged.

Regarding the safety of the method, there were no reports of serious adverse events related to the stimulation. Minor side effects were described in two studies, namely: reversible unilateral blurred vision, headache, neck pain, and drowsiness.

The use of rTMS has already been shown to be effective in the management of depression and anxiety after stimulation of the DLPFC. In this review, two studies demonstrate improvement in symptoms related to anxiety and depression after stimulation of other cortical areas such as the M1 motor cortex and SMC, although no conclusive evidence can be found in this subgroup of patients. Further studies that evaluate other cortical targets should be encouraged, as they may also demonstrate benefits in the management of mood changes.

There are some limitations to this study. The number of studies that evaluated the use of rTMS in the management of PLP is very small. In the last seven years, only two randomized clinical trials have been published. The additional data comes from case reports or estimates based on other painful conditions. As is known, chronic pain is a broad and heterogeneous condition, being subdivided into several syndromes and subcategories and presenting different responses to treatment in each of them. Another important factor is the non-standardization between the studies of the modulation parameters used. Although high-frequency stimulation in M1 is widely used, other parameters such as intensity, number of induction and maintenance sessions, and number of pulses are usually not standardized. Such parameters are also extremely important for the effectiveness of the therapy. The results of the studies also do not allow conclusions about the long-term analgesic effect of rTMS, given that most studies only evaluated the short-term response and did not perform a follow-up over a longer period of time. It should be noted that studies also do not usually describe pharmacological and non-pharmacological therapies that are being used concomitantly with stimulation. The evaluation of these parameters is important, since in clinical practice, rTMS is not usually used as a single therapy in the treatment of painful processes but is associated with other therapies.

The paucity concentration of studies related to the use of rTMS in the management of PLP, associated with the small number of patients recruited in these studies (most of them being case reports) and also the lack of standardization of the different stimulation parameters used in each one of these works are limiting factors that prevent an effective comparison and a global evaluation of the use of the method for this purpose. The results presented, although most are positive, cannot be generalized.

In conclusion, all studies evaluated in this review indicated some benefit, even if temporary, in the use of rTMS to relieve painful symptoms in patients with PLP. The maintenance of this analgesic effect varied between studies, with no study demonstrating worsening of pain after the end of sessions. Among the parameters used, high-frequency stimulation at M1 demonstrated a significant analgesic effect. Other targets have already been studied and proved to be ineffective, and although in this review some suggest benefits, they do not have statistical relevance with the strength of recommendation. The intensity, the number of pulses, as well as the treatment time, still need to be better evaluated to determine the recommendation.

Given the potential that has been demonstrated, but limited by the paucity of high-quality studies that deeply investigate the parameters, safety, and efficacy of rTMS in the treatment of PLP, further controlled studies are needed to establish and standardize the clinical use of the method.

## References

[1] Raja S N Carr D B Cohen M The revised International Association for the Study of Pain definition of pain: concepts, challenges, and compromises Pain 2020 161 09 1976 1982 32694387 10.1097/j.pain.0000000000001939 PMC7680716

[2] Melzack R Katz J Pain Wiley Interdiscip Rev Cogn Sci 2013 4 01 1 15 10.1002/wcs.1201 26304172

[3] Baptista A F Fernandes A MBL Sá K N Latin American and Caribbean consensus on noninvasive central nervous system neuromodulation for chronic pain management (LAC _2_ -NIN-CP) Pain Rep 2019 4 01 e692 10.1097/PR9.0000000000000692 30801041 PMC6370142

[4] Akyuz G Giray E Noninvasive neuromodulation techniques for the management of phantom limb pain: a systematic review of randomized controlled trials Int J Rehabil Res 2019 42 01 1 10 30222617 10.1097/MRR.0000000000000317

[5] IASP.2014 Postamputation painAvailable at:https://www.aped-dor.org/images/FactSheets/DorNeuropatica/en/Postamputation_Pain.pdf

[6] Pacheco-Barrios K Meng X Fregni F Neuromodulation Techniques in Phantom Limb Pain: A Systematic Review and Meta-analysis Pain Med 2020 21 10 2310 2322 32176286 10.1093/pm/pnaa039 PMC7593798

[7] Nardone R Versace V Sebastianelli L Transcranial magnetic stimulation in subjects with phantom pain and non-painful phantom sensations: A systematic review Brain Res Bull 2019 148 1 9 30862485 10.1016/j.brainresbull.2019.03.001

[8] García-Pallero MÁ Cardona D Rueda-Ruzafa L Rodriguez-Arrastia M Roman P Central nervous system stimulation therapies in phantom limb pain: a systematic review of clinical trials Neural Regen Res 2022 17 01 59 64 34100428 10.4103/1673-5374.314288 PMC8451556

[9] Batsford S Ryan C G Martin D J Non-pharmacological conservative therapy for phantom limb pain: A systematic review of randomized controlled trials Physiother Theory Pract 2017 33 03 173 183 28339333 10.1080/09593985.2017.1288283

[10] Richardson C Kulkarni J A review of the management of phantom limb pain: challenges and solutions J Pain Res 2017 10 1861 1870 28860841 10.2147/JPR.S124664 PMC5558877

[11] Higgins J PT Thomas J Chandler J Cochrane Handbook for Systematic Reviews of Interventions version 6.3 (updated February 2022)Cochrane,2022. [date of access: November 2022]. Available at:www.training.cochrane.org/handbook

[12] Page M J A declaração PRISMA 2020: diretriz atualizada para relatar revisões sistemáticas Epidemiol Serv Saude 2022 31 02 1 20 10.1590/s1679-49742022000200033

[13] Lefaucheur J P André-Obadia N Antal A Evidence-based guidelines on the therapeutic use of repetitive transcranial magnetic stimulation (rTMS) Clin Neurophysiol 2014 125 11 2150 2206 25034472 10.1016/j.clinph.2014.05.021

[14] Ahmed M A Mohamed S A Sayed D Long-term antalgic effects of repetitive transcranial magnetic stimulation of motor cortex and serum beta-endorphin in patients with phantom pain Neurol Res 2011 33 09 953 958 22080997 10.1179/1743132811Y.0000000045

[15] Malavera A Silva F A Fregni F Carrillo S Garcia R G Repetitive transcranial magnetic stimulation for phantom limb pain in land mine victims: A double-blinded, randomized, sham-controlled trial J Pain 2016 17 08 911 918 27260638 10.1016/j.jpain.2016.05.003 PMC4969102

[16] Scibilia A Conti A Raffa G Resting-state fMR evidence of network reorganization induced by navigated transcranial magnetic repetitive stimulation in phantom limb pain Neurol Res 2018 40 04 241 248 29380683 10.1080/01616412.2018.1429203

[17] Grammer G G Williams-Joseph S Cesar A Adkinson D K Spevak C Significant reduction in phantom limb pain after low-frequency repetitive transcranial magnetic stimulation to the primary sensory cortex Mil Med 2015 180 01 e126 e128 25562869 10.7205/MILMED-D-14-00236

[18] Lee J H Byun J H Choe Y R Lim S K Lee K Y Choi I S Successful treatment of phantom limb pain by 1 Hz repetitive transcranial magnetic stimulation over affected supplementary motor complex: a case report Ann Rehabil Med 2015 39 04 630 633 26361601 10.5535/arm.2015.39.4.630 PMC4564712

[19] Di Rollo A Pallanti S Phantom limb pain: low frequency repetitive transcranial magnetic stimulation in unaffected hemisphere Case Rep Med 2011 2011 130751 21629848 10.1155/2011/130751 PMC3099190

[20] Zhang K L Yuan H Wu F F Analgesic Effect of Noninvasive Brain Stimulation for Neuropathic Pain Patients: A Systematic Review Pain Ther 2021 10 01 315 332 33751453 10.1007/s40122-021-00252-1 PMC8119533

[21] Hirayama A Saitoh Y Kishima H Reduction of intractable deafferentation pain by navigation-guided repetitive transcranial magnetic stimulation of the primary motor cortex Pain 2006 122(1-2):22 27 10.1016/j.pain.2005.12.001 16495011

[22] Makin T R Filippini N Duff E P Henderson Slater D Tracey I Johansen-Berg H Network-level reorganisation of functional connectivity following arm amputation Neuroimage 2015 114 114 217 225 25776216 10.1016/j.neuroimage.2015.02.067 PMC4461307

[23] Makin T R Scholz J Filippini N Henderson Slater D Tracey I Johansen-Berg H Phantom pain is associated with preserved structure and function in the former hand area Nat Commun 2013 4 1570 23463013 10.1038/ncomms2571 PMC3615341

